# Predoctoral grant writing among physician-scientist trainees: a national survey of barriers and facilitators of success

**DOI:** 10.1172/jci.insight.208965

**Published:** 2026-09-01

**Authors:** Brian J. Thomas, Tiger Zhang, Daniel C. Brock, Timothy J. Ley, W. David Arnold, Cynthia Y. Tang

**Affiliations:** 1 Tom and Anne Smith MD-PhD Program, University of Missouri School of Medicine, Columbia, Missouri, USA.; 2 American Physician Scientists Association, Westford, Massachusetts, USA.; 3 University of North Carolina School of Medicine, Chapel Hill, North Carolina, USA.; 4 Medical Scientist Training Program, Baylor College of Medicine, Houston, Texas, USA.; 5 Division of Oncology, Department of Medicine, Washington University, St. Louis, Missouri, USA.; 6 Department of Physical Medicine and Rehabilitation, University of Missouri, Columbia, Missouri, USA.; 7 Department of Anesthesiology, Academic Career Enrichment Scholars Program, Duke University Medical Center, Durham, North Carolina, USA.

## Abstract

Effective grant writing is an essential skill for physician-scientists to achieve academic independence and long-term career success. Previous studies have established that receiving an NIH F30 or F31 during predoctoral training is correlated with success in subsequent training stages and contributes to the retention of physician-scientists in academia. However, many trainees experience challenges in predoctoral grant writing that prevent them from submitting a grant or developing a well-rounded application. Identifying and addressing such barriers and obstacles remains crucial; however, limitations in NIH public reporting exclude data on prospective applicants and applicants who were not awarded grants. In this study, we employed a national survey of trainees to identify perceived needs and barriers to grant writing as well as factors associated with NIH predoctoral grant funding success. We found that limited mentor and sponsor support of the development of quality applications, constrained eligibility timelines, and limited available awards were prominent barriers to submission, while access to previously funded applications was the most valued resource among respondents. Using these findings, we highlight opportunities for interventions at the federal, institutional, applicant, and medical and scientific society levels to improve predoctoral grant writing feasibility and success.

## Abstract 2

Publically available data and a survery were utilized to identify barriers to and challenges experienced during grant writing for dual-degree trainees across the nation.

## Graphical abstract

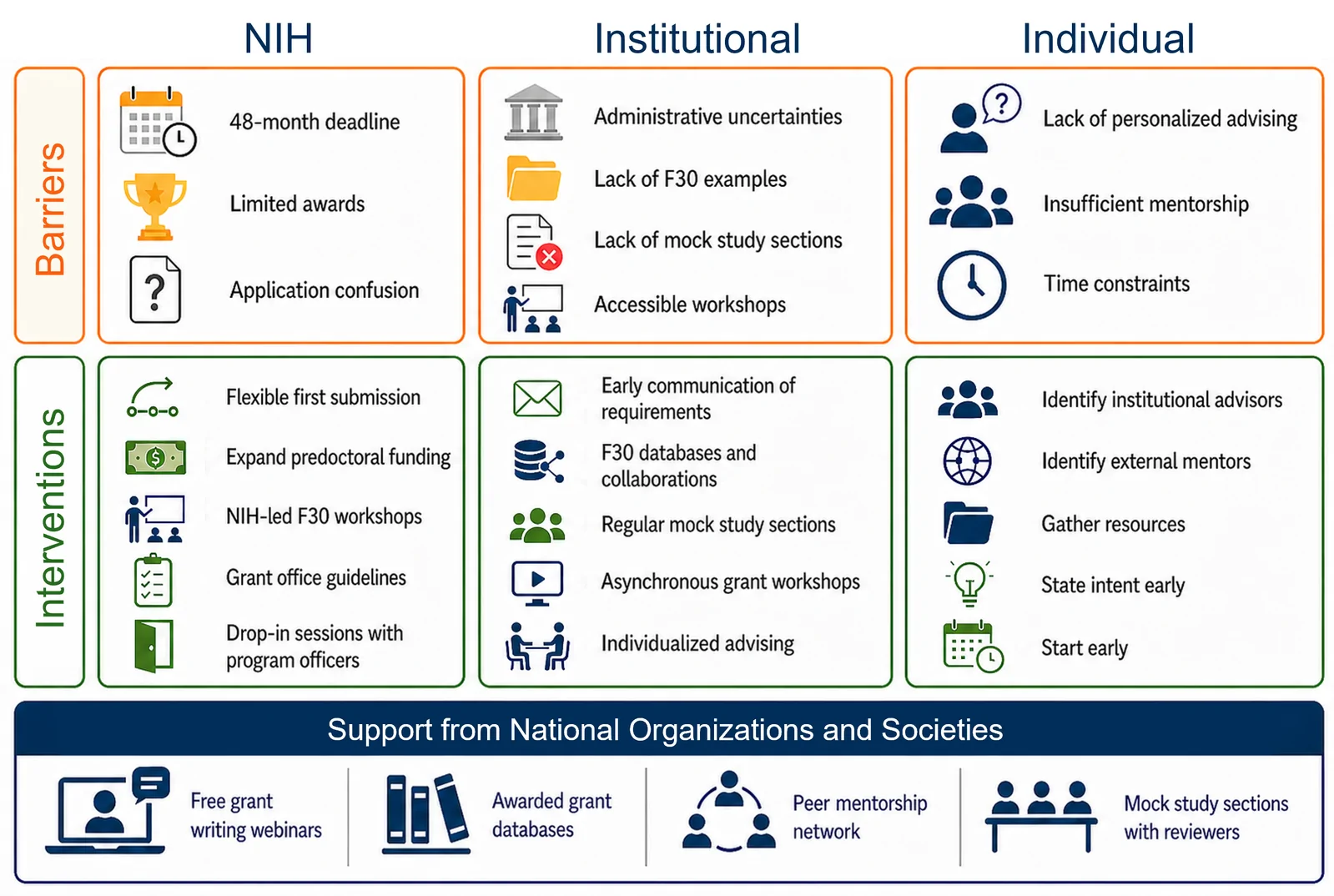

## Introduction

Physician-scientists play a critical role in driving medical advancements. However, the proportion of physicians who reported research as a major professional activity declined to 1.3% in 2020 (1). Attrition remains high at each transitional stage of physician-scientist training (2), and interest in research careers among graduating medical students has decreased (1). Multiple factors contribute to this erosion of the workforce, including increasing clinical demands, prolonged training durations, and limited research funding as well as an increasing time to first NIH R01 grant (3–6). These challenges heighten vulnerabilities to attrition throughout physician-scientist careers.

A critical, addressable leverage point early in this career path is grant writing. For predoctoral trainees, grant writing is both a key developmental skill and a gateway to future career success. NIH F-series (i.e., F30 and F31) fellowships constitute central predoctoral funding mechanisms for dual-degree trainees. Early grant writing success benefits research-track residency applications (7) and eventual R01 funding (6, 8), highlighting the reinforcing nature of early funding: initial success increases future funding opportunities and career progression. Despite its importance, grant writing during predoctoral training remains challenging. The publicly available NIH RePORTER database does not distinguish among degree programs, report applicant demographics, or include data on applicants who did not receive an award. Thus, the current literature primarily analyzes awardees rather than prospective applicants or applicants who applied and did not receive funding. Consequently, there is limited understanding about barriers to submission and obstacles to grant writing. As NIH budgets for training mechanisms face increasing pressure, this lack of understanding further limits efforts to sustain the physician-scientist workforce (4).

This study aims to address our limited understanding of the predoctoral grant writing process by employing a national survey of current and past MD/PhD or DO/PhD trainees, regardless of whether they had submitted a grant. This trainee-provided feedback allowed us to assess various aspects of predoctoral grant writing including (a) perceived benefits and obstacles to grant writing for those who submitted or planned to submit, (b) perceived barriers for those who did not submit or resubmit, (c) resource availability and perceived utility, and (d) perceived application strengths and weaknesses stratified by award status. These survey data were supplemented with analyses of publicly available data to investigate broad trends in predoctoral grant applications and funding, which demonstrated a decreasing trend in awards despite increasing interest. The findings of this study serve to identify gaps and direct targeted interventions to improve access to obtaining predoctoral grants, guide future applicants, and preserve the physician-scientist workforce.

## Results

### Study population.

We received 342 total responses to the survey. Nine were excluded owing to lack of consent or ineligibility for applying for an F30/F31 grant. Incomplete and nonsensical responses were excluded, leaving 256 complete responses. Survey census and demographic data are provided in Table 1 and Supplemental Table 1 (supplemental material available online with this article; https://doi.org/10.1172/jci.insight.208965DS1). Of the 256 responses included in the analyses, 46% (119 of 256) had not yet submitted a grant and 54% (137 of 256) had submitted a grant (117 F30s, 20 F31s). Of those who had submitted, 12% (16 of 137) did not receive an award and did not resubmit, 44% (60 of 137) did not receive an award but resubmitted or were planning to resubmit, and 45% (61 of 137) received an award either during their first submission or resubmission. Of respondents, 73% (187 of 256) were part of an NIH-funded medical scientist training program (MSTP), and 27% (69 of 256) were part of a non-NIH-funded MD/DO-PhD program (Table 1). The distribution of responses of participants from an MSTP and non-MSTP is broadly consistent with the national MD-PhD trainee population (ref. 9; 2025 AAMC FACTS, ref. 10). The overall demographics of the study population were similar to those of previous reports (11) and for current dual-degree matriculants (10). There were no demographic differences among those who submitted and those who did not submit a grant. Furthermore, there was no difference in demographics between those who submitted and were awarded a grant versus those who did not receive an award (Supplemental Table 1).

### Interest and motivations for predoctoral grant writing.

While MD/DO-PhD trainees are eligible to apply for both NIH F30 and F31 grants, the F30 grants specifically serve to enhance integrated research and clinical training of those who wish to have careers as physician-scientists (https://grants.nih.gov/). Analysis of publicly available data through NIH RePORTER (https://reporter.nih.gov/) demonstrate that the number of F30 applications have risen by 39% from 545 in 2017 to 760 in 2025 (Figure 1A). When compared with a 13% increase in total student enrollment over this period (5,408 in 2017 to 6,159 in 2025; ref. 10), the size of the increase suggests an increasing proportion of F30 applicants (Supplemental Figure 1A). Despite the increasing percentage of applicants, the number of awards has remained relatively constant since 2017; consequently, success rates have declined from a high of approximately 42% in 2018 to a low of 32% in 2025 (Figure 1A). For F31 grants, applications and awards have generally shown an appreciable and equitable rise since 2018; however, they exhibited a sharp decline in awards in 2025, leading to a decade’s low success rate of 23.5% (Supplemental Figure 1B; NIH RePORTER).

To understand the motivations of applicants and potential applicants, we assessed the perceived benefits for applying for NIH predoctoral grants (Figure 1, B and C). Of survey respondents, 230 ranked 4 predefined perceived benefits of applying for an F award from 1, signifying no benefit, to 4, signifying significant benefit. Of these, 95% (218 of 230) identified grant writing experience, 95% (219 of 230) identified establishing a funding track record for future grants, 94% (217 of 230) identified strengthening their CV for their future career, and 73% (168 of 230) identified financial support as either a moderate or significant benefit. These self-identified benefits were perceived similarly between the submission and award groups (Figure 1B). In addition, 35 individuals provided free-text responses describing additional benefits to grant writing. Of these, 24% (10 of 35) noted improvement in their dissertation and PhD efficiency, 14% (6 of 35) indicated an additional institutional allowance or stipend bonus, 12% (5 of 35) noted grant writing experience for an academic career, 9.5% (4 of 35) found increased confidence, 7.1% (3 of 35) indicated expanding their network, and 7.1% (3 of 35) used the process as preparation for their qualifying exam. Others noted that grant writing provided inspiration for considering nonacademic career paths, status and prestige, demonstration of the value of their research, increased knowledge of their research subject, increased competitiveness for residency, opportunity to extend their PhD training to complete a more robust project, healthcare coverage, personal growth, and increased subject knowledge. Three respondents noted that submitting an F30 was a program requirement (Figure 1C).

### Barriers to predoctoral grant submission and resubmission.

Despite these reported benefits, NIH predoctoral grants often take an extensive amount of time, effort, and guidance to complete, requiring collaboration among the applicant, the mentoring team, and the institution. To identify barriers that limit grant submission or resubmission, we asked survey respondents who did not plan to apply to or resubmit a predoctoral grant to identify their reasons, using a prespecified list (Figure 2A). Of 26 respondents who noted that they did not plan to apply at all, 19% (5 of 26) identified structural factors, including not having enough resources (8%, 2 of 26) or an unsupportive mentor (8%, 2 of 26). Capacity and effort constraints were noted in 38% (10 of 26) of responses, including not having enough time to complete a proposal (27%, 7 of 26). Eligibility was noted in 57% (15 of 26) of responses, including being too late to submit because of the 48-month postmatriculation limit (23%, 6 of 26) or other reasons for being ineligible (34%, 9 of 26). Personal factors were noted in 47% (12 of 26) of responses, including already having other funding or scholarships (31%, 8 of 26).

Of 16 respondents who applied but were not awarded and noted they did not plan to resubmit, 50% (8 of 16) identified structural factors, 69% (11 of 16) identified capacity and effort constraints, 44% (7 of 16) identified eligibility, and 81% (13 of 16) identified personal factors. For these potential reapplicants, the most common reasons provided in free-text responses for not applying included not having enough time to complete a proposal (63%, 10 of 16), already having other funding (56%, 9 of 16), having an unsupportive mentor (38%, 6 of 16), or being too late to resubmit (44%, 7 of 16).

### Challenges during predoctoral grant writing.

To identify obstacles limiting successful grant writing, respondents were asked to rank 4 predefined perceived obstacles from 1, signifying not an obstacle, to 4, signifying a significant obstacle (Figure 2B). Of 230 respondents who either submitted or were planning to submit a predoctoral grant, 75% (173 of 230) identified the effort required to complete the grant, 26% (60 of 230) identified finding collaborators, 23% (55 of 230) identified sponsor or cosponsor support, and 23% (54 of 230) identified access to resources as either a moderate or significant obstacle. Compared with those who submitted a grant, a higher percentage of those who planned to but had not yet submitted identified finding collaborators (40%, 37 of 93 vs. 17%, 23 of 137; *P* = 0.0002) as either a moderate or significant obstacle. Of applicants who submitted, compared with those who received an award, those who did not receive an award had a higher percentage that identified sponsor or cosponsor support (29% vs. 9.8%; *P* = 0.011) as either a moderate or significant obstacle.

Of the 230 respondents, 64 reported additional perceived obstacles in free-text format (Figure 2C). The most reported obstacle was time limitation (22%, 14 of 64), where respondents felt constrained by the 48-month window to first submission (9.4%, 6 of 64), often citing insufficient timing to generate preliminary data, particularly for course-heavy PhDs or programs that complete 3 preclinical/clinical years prior to beginning the PhD. They also cited the time required to assemble the grant application (15.4%, 10 of 64) as a barrier that took away time from other responsibilities. The second most common barrier was lack of mentorship (26.6%, 17 of 64), which included lack of mentor time, support, or feedback; lack of mentor experience or success with MD/PhD or DO/PhD trainees or writing NIH grants; and lack of grant writing–specific mentorship. Third was a lack of institutional support (21.9%, 14 of 64), which included lack of supplemental tuition beyond the 60% provided by the F30, lack of institutional grant writing courses, not having a track record of awarded F30s, being a smaller program, lack of examples from prior applicants, and, most commonly reported, challenges with internal administrative offices (20.3%, 13 of 64). Additional barriers included lack of NIH communication and resources (10.9%, 7 of 64), application components (10.9%, 7 of 64), understanding the grant process (6.3%, 4 of 64), and personal wellbeing (3.1%, 2 of 64). Reported lack of NIH resources included limited funding opportunities, funding uncertainty, and difficulty contacting the NIH for information and feedback. For application components, respondents listed preliminary data (6.3%, 4 of 64), research plan, and lack of a personal research record as obstacles.

### Predoctoral grant writing resource availability and utility.

To identify resource utility, survey respondents were asked what resources were offered and used, what resources were the most useful during grant writing, and what resources they wished they had access to (Figure 3). Of 230 respondents who either submitted or were planning to submit a predoctoral grant, 204 answered questions about resources that were available or that were used (Figure 3A). Overall, 74% (151 of 204) of respondents reported that their programs or institutions offered grant writing workshops, 75% (153 of 204) offered grant writing information sessions, 52% (106 of 204) offered additional advising, 41% (84 of 204) offered mock review sessions, and 84% (171 of 204) offered example successful grants. When compared with other resources available at their institution (e.g., graduate schools), respondents reported their dual-degree programs had higher rates of offering successful example grants (81%, 165 of 204 vs. 29%, 59 of 204), grant writing information sessions (59%, 120 of 204 vs. 38%, 78 of 204), and additional advising (41%, 84 of 204 vs. 23%, 47 of 204).

In terms of resources used by applicants, successful example grants were the most often used resource (69%, 141 of 204), followed by information sessions (59%, 120 of 204), grant writing workshops (50%, 102 of 204), additional advising (37%, 75 of 204), and mock review sessions (27%, 54 of 204). Respondents overwhelmingly used resources available through their program or institution over external resources, with successful example grants being the most used external resource (20%, 41 of 204). Few applicants reported access to no resources (1%, 2 of 204) or used no resources (10%, 20 of 204), and 51% (104 of 204) did not use any external resources. Of 230 respondents who either submitted or were planning to submit a predoctoral grant, 137 also answered questions about peer access; 96% (132 of 137) had access to successfully funded peer applications, while 99% (136 of 137) had access to any peer resource.

These 204 respondents also answered questions about resources they found the most useful and resources that were desirable but unavailable (Figure 3B). For usefulness, successful grants were the most common reported resource (63%, 129 of 204), followed by grant writing workshops (30%, 61 of 204) and grant writing information sessions (22%, 45 of 204). For desirable but unavailable resources, mock review sessions were the most commonly selected (38%, 78 of 204), followed by additional advising (26%, 53 of 204) and grant writing workshops (24%). Of the 204 respondents, 67 provided additional free-text feedback on resources that were used, and 52 respondents provided additional free-text input on desirable but unavailable resources (Figure 3C). These responses largely aligned with the checkbox data above, with a focus on mentoring and advising resources, mock study sections, and access to successful applications, particularly in the NIH center that they were applying to and for nonbasic science projects. Notably, 9% of free-text respondents directly mentioned the book, *A Practical Guide to Writing a Ruth L*. *Kirschstein NRSA Grant*.

### Perceived application strengths and weaknesses.

Grant applicants were also asked about the perceived strengths and weaknesses of their application. Respondents who were *awarded* an NIH predoctoral grant were given the opportunity to provide free-text responses to their perceived strengths of their applications (Supplemental Figure 2A). Of 61 respondents who received an award on first submission, 60 provided additional free-text responses. These respondents noted that their research plan (35%, 21 of 60), which included impact, novelty, and strong preliminary data; applicant profile (35%, 21 of 60), which included their narrative and background, strong academic performance record, and publication record; mentor team (33%, 20 of 60); training plan (17%, 18 of 60); and institutional support/environment (10%, 6 of 60) were strong in their applications. Writing and formatting skills were noted by 8% (5 of 60). For resubmissions, 24 of 25 respondents who were ultimately awarded provided free-text responses regarding what the biggest driving factor was to turn their resubmission into a successful application. Of these, 46% (11 of 24) attributed their strengths to their improved research plan, 29% (7 of 24) noted strong preliminary data, 25% (6 of 24) noted mentorship and improved mentor team, 17% (4 of 24) noted improved training plan, and 29% (7 of 24) reported addressing all reviewer comments as a driving factor to success.

Respondents who submitted an NIH predoctoral grant, regardless of award, were also given the opportunity to provide free-text responses to the perceived pitfalls of their applications (Supplemental Figure 2B). Overall, of first-time submitters, 117 respondents attributed their pitfalls to their research strategies and aims (55%, 65 of 117), which included a poorly developed research plan and aims (26%, 33 of 117), insufficient preliminary data (7.4%, 10 of 117), and overly ambitious aims/scope (12%, 13 of 117). Lack of mentorship (26%, 30 of 117) was the next most cited pitfall, which involved the sponsor lacking experience with training dual-degree students, mentor lack of funding, and overall inadequate mentorship. Insufficient training plans were attributed by 14% (19 of 117) of respondents. 11% (13 of 117) of respondents received feedback that their applicant profile was weak, with 8.1% (10 of 117) noting limited or no prior publications. Compared with the 60 applicants who received an award on first submission, applicants who did not receive an award described pitfalls involving mentorship at a higher frequency (37%, 21 of 57 vs. 15%, 9 of 60; *P* = 0.013), including noting a lack of mentor funding (12%, 7 of 57 vs. 0%, 0 of 60; *P* = 0.005). Perceived pitfalls from 32 respondents who resubmitted and were not awarded were also assessed. Responses aligned with their first submission pitfalls with slightly less emphasis on mentorship.

## Discussion

Predoctoral funding plays a pivotal role in shaping both the trajectory of physician-scientist careers and the future landscape of biomedical discovery. Using publicly available data and a national survey of dual-degree trainees, this study revealed predoctoral grant application and funding trends, perceived benefits and obstacles to grant writing, perceived barriers to grant submission, grant writing resource access and perceived utility, and application factors associated with an awarded grant. Responses from applicants as well as from applicants considering future submission allowed us to capture a more generalizable picture of perceived benefits and barriers relative to publicly available data alone and allowed us to compare responses among these groups. Overall, our findings reveal opportunities for tangible interventions that may improve the predoctoral grant writing experience.

Nationally, there is increasing interest in F30 awards, demonstrated by an increasing number of NIH predoctoral grant applications (Figure 1A and Supplemental Figure 1) and clear perceived benefits of these awards (Figure 1, B and C). Despite this increase, many dual-degree trainees still do not apply. Our findings demonstrate that this is largely due to time or effort limitations, eligibility, and mentorship (Figure 2A). We also speculate that the limited number of available awards, despite increased application numbers, contributes to worsening funding success rates, which may discourage prospective applicants. This problem may be further compounded by recent government funding cuts (4, 12). For those who do apply, many trainees face challenges in developing quality applications. Respondent-reported obstacles and pitfalls highlighted underdeveloped research plans, lack of adequate mentorship, and limited resources as contributors (Figure 2, B and C, and Supplemental Figure 2B). As research plan weaknesses can often be addressed by proper mentorship, it seems increasingly important that finding appropriate mentors and defining how these mentors will allow a trainee to achieve their research plan (i.e., a training plan) is necessary for predoctoral grant writing success. Overall, we suggest collaboration between the applicant, a strong mentoring team, the applicant’s program and institution, and equitable accessibility of resources is of utmost importance for writing a compelling application.

We also compared applicant professional experience, institutional environment, and mentor/sponsor background of applicants who received a predoctoral award and those who did not. We found that very few factors showed statistically significant associations for being awarded a predoctoral grant (Supplemental Figure 3). This relative lack of background-based disparities in award outcomes should be encouraging for those who wish to apply. This, along with our survey-identified strengths of successful applications (Supplemental Figure 2A) and valued resources (Figure 3) may serve as a guide for future predoctoral grant applicants.

Nevertheless, applicants still face many challenges, as described above, and our findings lead us to recommend the following practical interventions to help alleviate these trainee-perceived barriers and obstacles.

At the federal level (i.e., NIH), eligibility was frequently noted as a barrier. The 48-month restriction to first submission for F30s, and the requirement of needing at least equal or greater time left in PhD training compared with medical training, greatly limit applicant eligibility. These two restrictions are particularly problematic for programs or individuals that pursue the 3-4-1 and 2-4-2 (preclinical-PhD-clinical) pathways, respectively. To address eligibility limitations, the NIH should consider increasing flexibility for the submission timeline. Additionally, our analysis of public NIH data also points to a flat number of awards with declining success rates over the past decade. As predoctoral funding allocations are only a small fraction of the federal budget, yet the resulting medical advancements and improved health outcomes offer a substantial return on investment (4, 13), the NIH should consider expanding investments in dual-degree training grants.

At the institutional (school or program) level, respondents noted a lack of helpful resources. Institutions should establish databases of awarded dual degree-specific fellowships, collaborate with other institutions to access successful examples when such examples are not available in-house, provide individualized advising to both trainees and new MD/DO-PhD mentors emphasizing the role of the mentoring team in helping the trainee develop skills to achieve feasible goals proposed in a research plan (14), and offer mock study sections to provide grant feedback and allow trainees to experience the evaluation process.

At the applicant level, many respondents noted lack of adequate mentorship as a notable barrier or obstacle. Trainees can work with their program advisors to communicate their interest in submitting a fellowship early and utilize advisors and prior trainee experiences to identify a strong sponsor/mentor team. Students at institutions with limited experience training physician-scientists can work with their program advisors to identify additional mentors at other institutions who can provide feedback on training and research plans. Prospective applicants may also utilize external resources such as national grant writing webinars, *A Practical Guide to Writing a Ruth L*. *Kirschstein NRSA Grant*, and the Grant Writing Database curated by the American Physician Scientists Association (APSA).

Finally, national organizations and societies can help supplement institutional and trainee knowledge and alleviate gaps in resources. For example, the APSA (15, 16) has developed an extensive and growing database of grant writing resources, a peer mentoring network, and a series of virtual grant writing webinars. Notably, few respondents utilized resources provided from outside of their institution or programs (Figure 3A), possibly because they are uncommon or trainees are not aware of their availability. Continuing to build these resource databases and increasing awareness of their existence is necessary in the ever-evolving scientific landscape.

This study has several limitations. First, the response rate was about 4%, given there are over 6,000 dual-degree matriculates. A larger sample size would improve the margin of error. Second, to preserve anonymity, institutional identifiers were not collected with the survey; therefore, the representativeness and generalizability of our findings across regions or institutions are unknown. However, census and demographic data obtained from survey respondents (Table 1 and Supplemental Table 1) were largely similar to previous reports and current national data (Methods). This supports the representative nature of the study population. Third, survey data are subject to response bias. To mitigate this, we offered raffle-based compensation and emphasized that participation did not require prior submission or receipt of an award. Our observed F30 success rate (43.6%) should be interpreted alongside NIH-reported fiscal year 2024 success rates of 35.4% for F30 applications (715 total applications; https://report.nih.gov/). Fourth, we did not evaluate primary application materials or raw impact scores; we were therefore unable to directly assess the quality of applications. Fifth, our survey was primarily completed by MD-PhD trainees and F30 applicants, with insufficient representation of DO-PhD students and F31 applicants. As NIH RePORTER data do not differentiate applicant type (i.e., dual-degree versus PhD), it was not possible to assess whether our survey was representative nor was it possible to determine MD/DO-PhD–specific success rates for F31s (Supplemental Figure 1B). For reference, survey data specific to F31 grants was extracted and is provided in the Supporting Data Values file. Sixth, diversity mechanisms (e.g., F31D) were not assessed, potentially further limiting accurate capture of F31 applicants and those who are underrepresented in medicine. Finally, of note, major categorical changes were made to predoctoral grant submissions in early 2025 (https://grants.nih.gov/, Supplemental Figure 4) in an effort to minimize disparities from the suggested overinfluence of institutional status, mentor status, and applicant profile. Instead, the new stated goal is to increase focus on the training plan and whether the mentoring team is sufficient to complete the proposed research and whether the applicant is trainable. Future studies will be needed to determine if indeed these changes adequately address such disparities.

In conclusion, predoctoral funding enables scientific progress, signals preparedness for rigorous research pathways, and serves as a critical inflection point that can influence trainees’ access to opportunities and future success as physician-scientists. Responses from trainees suggest that structured institutional training improves predoctoral grant writing success (17), yet we and others (15) note that the quality of curricula remains inconsistent across programs. Ultimately, it will be the concerted efforts of the government, institutions, trainees, and societies that will help sustain interest in predoctoral grant writing and reduce attrition from the physician-scientist pipeline.

## Methods

### Survey data collection

Data were collected using a self-administered, structured online survey developed by the study team to assess challenging and contributing factors to success in predoctoral grant writing. The survey consisted of 188 items organized into 6 sections covering (a) grant submission(s) and outcomes; (b) resources, utilization, and perceptions; (c) applicant information and experience; (d) sponsor/cosponsor information; and (e) self-reported demographic questions. Quantitative (i.e., multiple choice, checkboxes, 4-point Likert-scale items, etc.) and qualitative (i.e., open-ended questions) items were featured throughout the survey (Supplemental Methods). For questions with prespecified responses, a group of 4 physician-scientist trainees at various institutions and stages of training who have applied for F30/F31 grants determined responses based on their experiences, and the consensus among all trainees was used for the survey.

The survey was administered electronically using the Research Electronic Data Capture (REDCap). Eligible participants included those who were either in an MD/DO-PhD program or had completed MD/DO-PhD training at a formal MD/DO-PhD program in the United States. Recruitment and survey distribution was achieved through the APSA using (a) emailed newsletters, (b) online community forums, (c) direct contact with APSA institutional representatives, and (d) direct contact with MD/DO-PhD program directors to maximize visibility to all current and past trainees.

Recruitment and data collection were performed from March 2024 through June 2025. Participation was voluntary, and informed consent was obtained electronically. Partially completed surveys were excluded from analysis. No personally identifying information was collected or linked to the survey record. Data were stored on secure, password-protected servers and were exported in deidentified form for analysis.

Compensation for participation included a raffle for 3 $50 e-gift cards. Each individual participating in the study had the option to provide their name and email address separately from the survey to be included in the raffle.

### Analysis of survey population representativeness

Post hoc analyses were performed to determine how well the study population represented the national trainee population. Given a large trainee population (i.e., greater than 5,000 grant-eligible trainees), the margin of error was calculated using a sample size of 256 complete surveys. Assuming a conservative sample proportion of 0.5, and a 95% confidence level, the survey yielded a margin of error of approximately 6.12% (18). To assess representativeness, census and demographic information such as program type (MSTP or non-MSTP), gender, ethnicity, parental education, and childhood household income was compared with that of previous studies (9, 11) or current national data (10). Census and demographic data in our study was largely similar to that of previously reported studies and current national data. In addition, emails, collected separately and voluntarily from those who participated in the raffle, included responses from at least 50 unique institutional email domains.

### Statistics

#### Quantitative data analysis.

All statistical analyses were performed in the R statistical suite (version 4.4.1). Statistical significance was defined using a threshold of α = 0.05. The proportions of survey responses across different subgroups (i.e., award versus no award, grant submission versus no submission) were compared with χ^2^ tests and Fisher’s exact tests when the number of responses within any subgroups was below 5. To analyze the association between survey variables and award status, univariable logistic regression was employed and the regression odds ratio was used to estimate the variable’s effect size. To correct for multiple hypothesis testing in logistic regression, Bonferroni’s correction was applied.

#### Qualitative data analysis.

All study participants were given the opportunity to provide additional context and insights through free-text responses throughout the survey. Researchers analyzing the free-text analyses included 2 PhD researchers who had previously applied for and received NIH F30s and 1 PhD student. Two researchers independently and manually reviewed and coded each response with hierarchical themes using Delve. Codes from both researchers were compared for agreement and only used for analysis if agreement was found. The third researcher served as a tiebreaker where needed. The number of responses associated with each theme were recorded.

#### National data analysis.

NIH RePORTER was used to determine the number of annual F30 and F31 applications reviewed, successful awards, and funding rates. Data for the total number of applications reviewed were available from 2015 to 2025. AAMC FACTS was used to determine the total number of MD-PhD enrollees and to compare dual-degree matriculant demographic information (10). Data were available from 2016 to 2025.

## Study approval

This study was determined to be exempt by the Institutional Review Board at the University of Missouri (IRB protocol 2097283).

## Data availability

Supporting data for all tables and figures are available in the Supporting Data Values file.

## Author contributions

BJT and CYT conceptualized the project. BJT, CYT, and WDA curated the data. BJT, CYT, and TZ were involved in methodology. BJT, CYT, and TZ wrote the original draft. DCB, TJL, and WDA edited the draft. CYT, TZ, and DCB analyzed the data. CYT supervised, administered, and obtained funding for the study. All authors were responsible for reviewing and editing the manuscript.

## Conflict of interest

The authors have declared that no conflict of interest exists.

## Funding support

This work is the result of NIH funding, in whole or in part, and is subject to the NIH Public Access Policy. Through acceptance of this federal funding, the NIH has been given a right to make the work publicly available in PubMed Central.

- APSA.
- NIH F30 grant through the National Cancer Institute (F30CA275349) to BJT.

## Supplementary Material

Supplemental data

Supporting data values

## Figures and Tables

**Figure 1:**
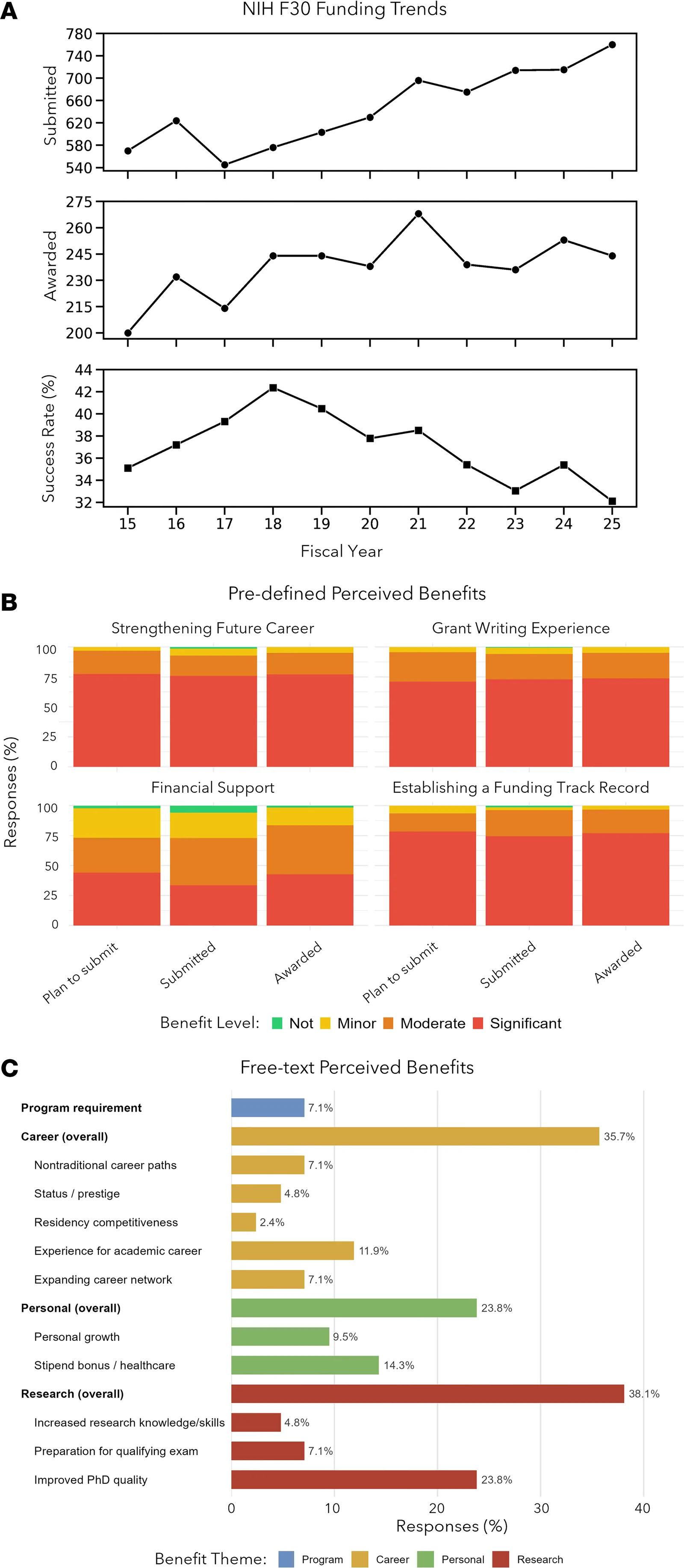
National NIH F30 trends and perceived predoctoral grant writing benefits. (**A**) Total number of NIH F30 grants submitted and reviewed, number of newly awarded F30 grants, and F30 success rate calculated as percentage of F30s awarded relative to those submitted by year (2015–2025). Fiscal year is reported on the *x* axis. Data were extracted from NIH RePORTER. (**B**) Applicant-perceived benefits of submitting a predoctoral grant from 4 predefined questions. Bars represent the percentage of respondents that reported a given benefit (*y* axis). Data are reported for those that submitted, planned to submit, or were awarded. (**C**) Free-text responses of applicant-perceived benefits. Subcategories are reported under major categories. Bars represent the percentage of respondents that reported a given benefit (*x* axis).

**Figure 2:**
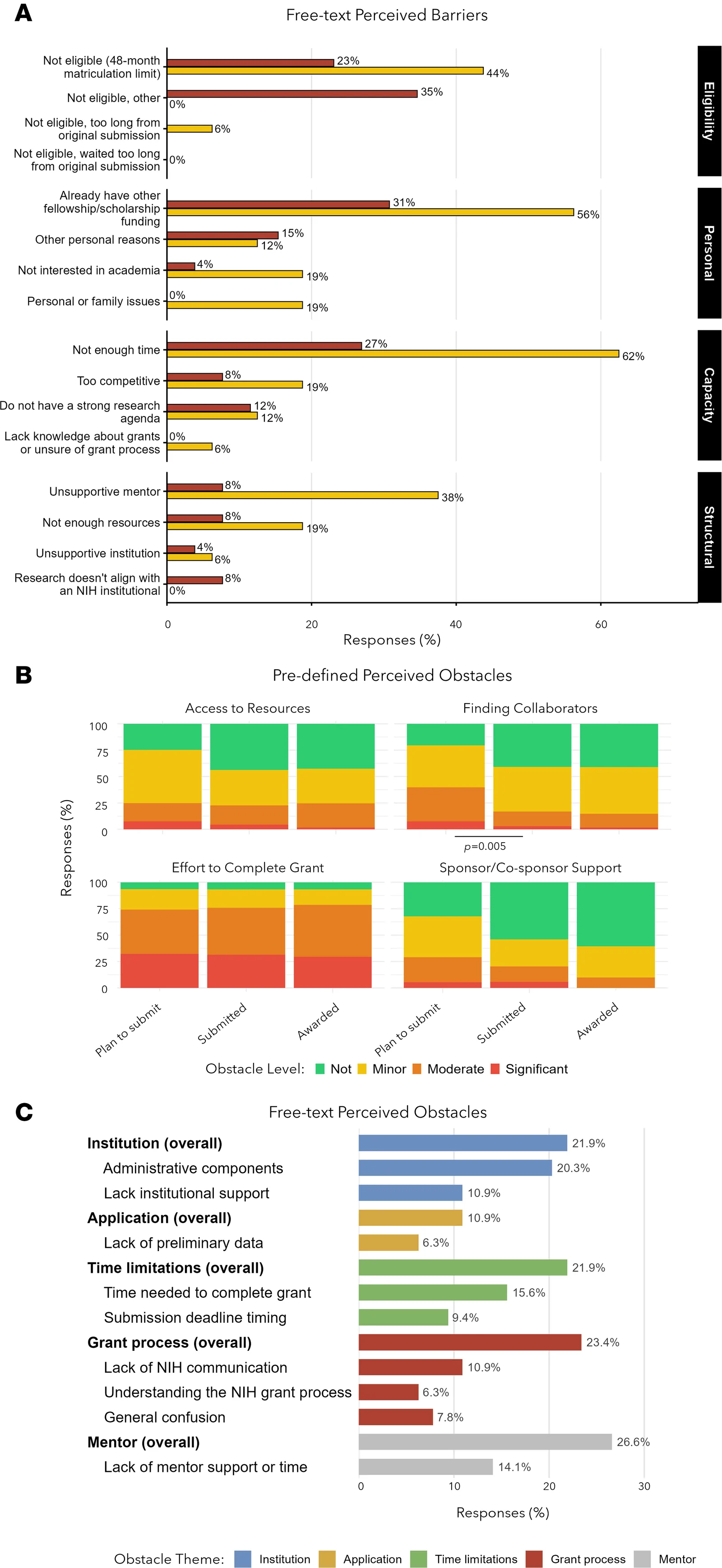
Perceived barriers to submission and obstacles to predoctoral grant writing. (**A**) Self-reported barriers that prevented grant submission or resubmission or (**B** and **C**) obstacles that presented difficulty during grant writing for those who submitted or planned to submit. (**A**) Free-text responses of applicant-perceived barriers to submission and resubmission by category. Red bar: no submission; yellow bar: no resubmission. (**B**) Applicant-perceived obstacles of submitting a predoctoral grant from 4 predefined responses. Bars represent the percentage of respondents that reported a given obstacle (*y* axis). Data are reported for those that submitted, planned to submit, or were awarded. Statistical analysis was performed using a χ^2^ test. (**C**) Free text responses of applicant-perceived obstacles.

**Figure 3:**
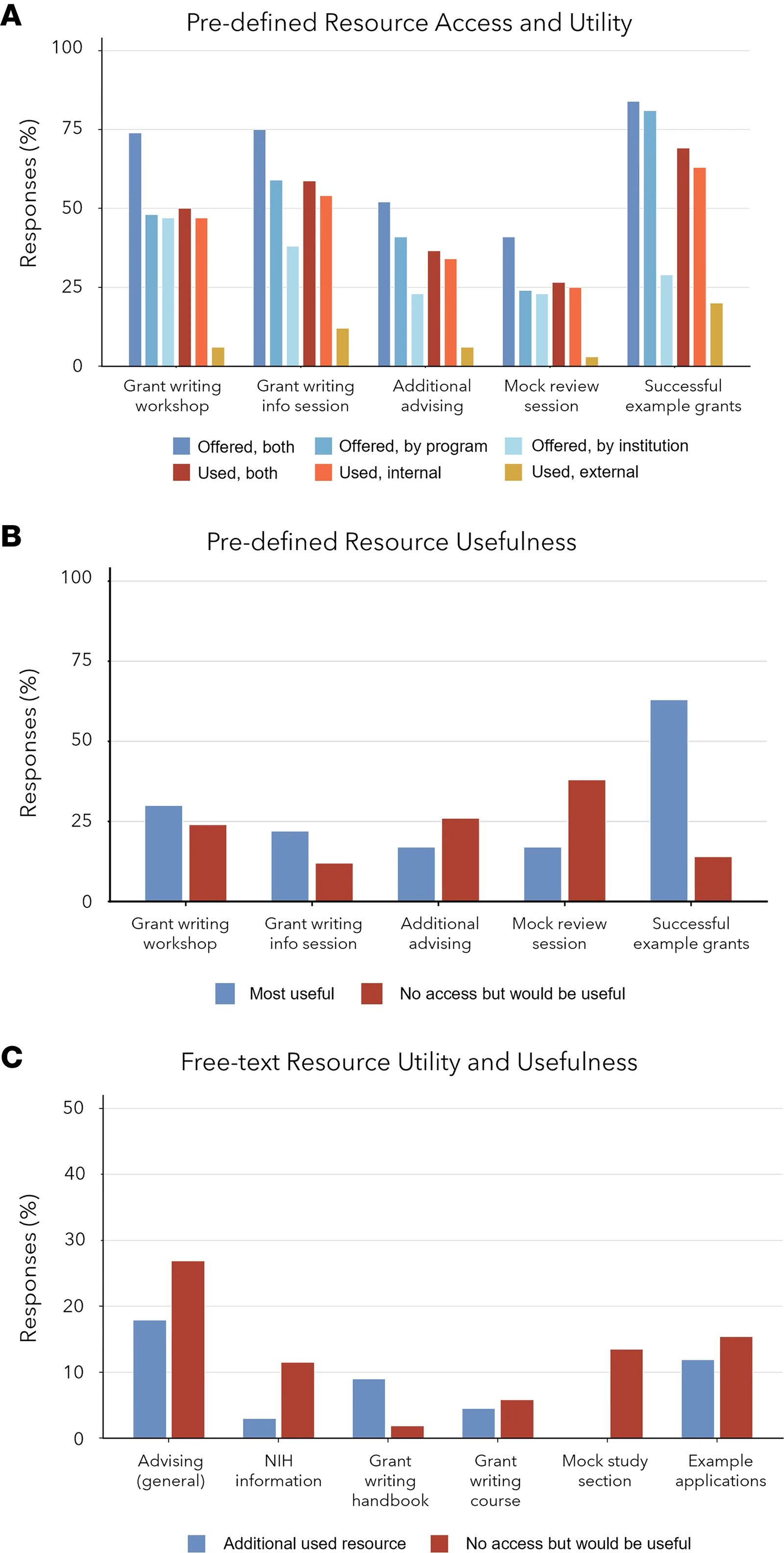
Resource utility and usefulness. Reported resource utility and usefulness for those who submitted or planned to submit a grant. (**A**) Resource availability offered by dual-degree program, institution, or both and utilization reported as internal, external (outside program or institution), or both. (**B**) Self-reported resource usefulness and unavailable resources that respondents felt would be useful. (**C**) Free-text responses of additional used resources and additional unavailable resources that respondents wish they had. Categories reporting ≥5 percent are reported. For all, resource type is reported on the *x* axis and the percentage of respondents is reported on the *y* axis.

**Table 1:**
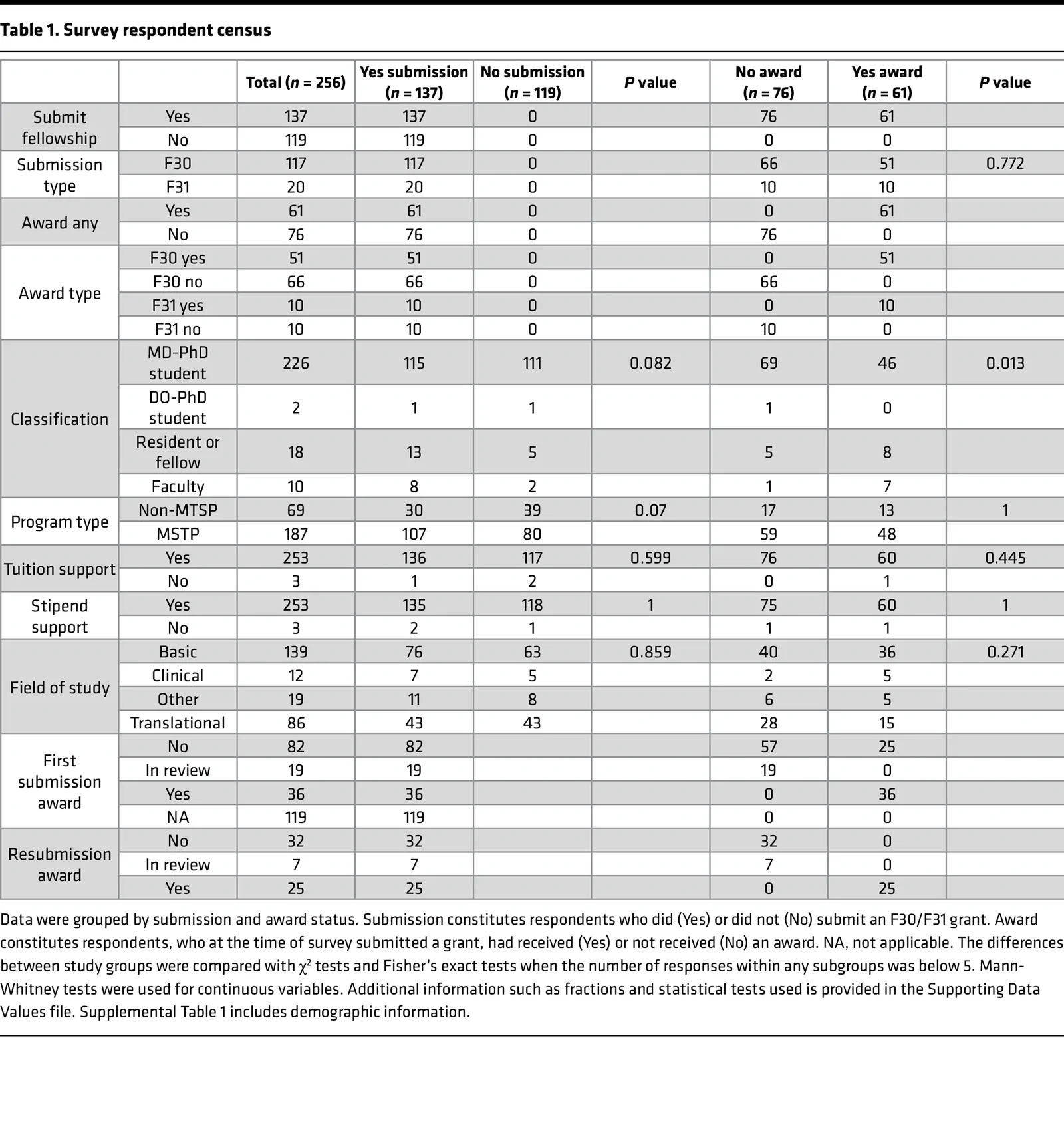
Survey respondent census

## Version 1 09/01/2026

In-Press Preview

## Version 2 10/08/2026

Electronic publication

## References

[1] Garrison HH, Ley TJ (2022). Physician-scientists in the United States at 2020: trends and concerns. FASEB J.

[2] Milewicz DM (2015). Rescuing the physician-scientist workforce: the time for action is now. J Clin Invest.

[3] Ley TJ, Hamilton BH (2008). Sociology. The gender gap in NIH grant applications. Science.

[4] Tang CY (2025). Training physician-scientists, a view from inside. Nat Med.

[5] Brass LF, Akabas MH (2019). The national MD-PhD program outcomes study: relationships between medical specialty, training duration, research effort, and career paths. JCI Insight.

[6] Ghosh-Choudhary S (2022). Predoctoral MD-PhD grants as indicators of future NIH funding success. JCI Insight.

[7] Gallagher EJ (2022). Pearls of wisdom for aspiring physician-scientist residency applicants and program directors. JCI Insight.

[8] Conte ML (2020). Trends in NIH-supported career development funding: implications for institutions, trainees, and the future research workforce. JCI Insight.

[9] Martinez-Strengel A (2022). Trends in U.S. MD-PhD program matriculant diversity by sex and race/ethnicity. Acad Med.

[10] https://www.aamc.org/data-reports/students-residents/data/facts-applicants-and-matriculants.

[11] Williams DKA (2024). Sociodemographic factors and research experience impact MD-PhD program acceptance. JCI Insight.

[12] Witze A (2025). US PhD admissions shrink as fears over Trump’s cuts take hold. Nature.

[13] Ayedun A (2023). Perspectives on National Institutes of Health funding requirements for racial and ethnic diversity among medical scientist training program leadership. JAMA Netw Open.

[14] Fitzsimonds RM (2024). Interventions to support fellowship application success among predoctoral physician-scientists. JCI Insight.

[15] Tang CY (2025). American Physician Scientist Association annual meetings: celebrating 20 years of physician-scientist training and collaboration. JCI Insight.

[16] Weng J (2025). 2024 Physician-Scientist Trainee Diversity Summit conference proceedings. J Clin Transl Sci.

[17] Souder JP (2022). A novel curricular framework to develop grant writing skills among MD-PhD students. J Clin Transl Sci.

[18] Serdar CC (2021). Sample size, power and effect size revisited: simplified and practical approaches in pre-clinical, clinical and laboratory studies. Biochem Med (Zagreb).

