# Supplementary material for: Predoctoral grant writing among physician-scientist trainees: a national survey of barriers and facilitators of success
Source: JCI Insight. 2026 Sep 1;11(19):e208965. doi: 10.1172/jci.insight.208965 (PMC13726420; doi:10.1172/jci.insight.208965)
Supplement: Supplementary file 1 [file jciinsight-11-208965-s096.pdf]

Supplemental Information

Figure S1: NIH F30 applicants by enrollment
(**A**) Number of total MD-PhD program enrollees and percentage of total MD-PhD trainees that apply for F30s by year (2016-2025). (**B**) Total number of NIH F31 grants submitted and reviewed, number of newly awarded F31 grants, and F31 success rate calculated as percentage of F31s awarded relative to those submitted by year (2015-2025). Data was extracted from NIH RePORTER and is not differentiated by applicant type (i.e., dual-degree versus PhD). For both, fiscal year is reported on the *x*-axis.

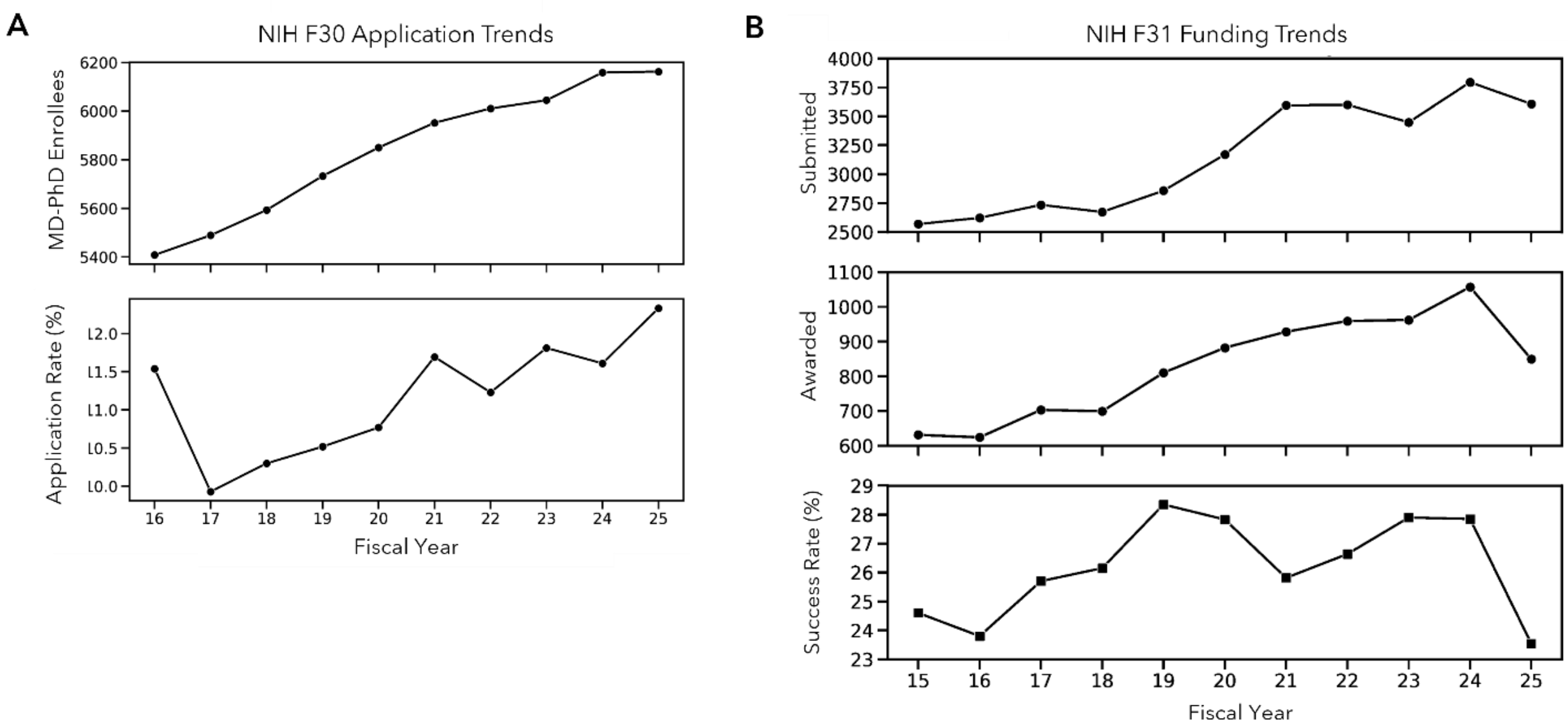

Figure S2: Application strengths and pitfalls.
(**A**) Free-text perceived application strengths from applicants who were awarded on first submission (dark shaded bars) or resubmission (light shaded bars). (**B**) Free-text perceived application pitfalls for those who were awarded (dark blue bar) or not awarded on first submission (light blue bar) or resubmission (orange bar). For both, percentage of respondents is reported on the *x*-axis. Major categories are reported on the *y*-axis in bold with sub-categories beneath. Statistical analysis was performed using a Chi-squared test.

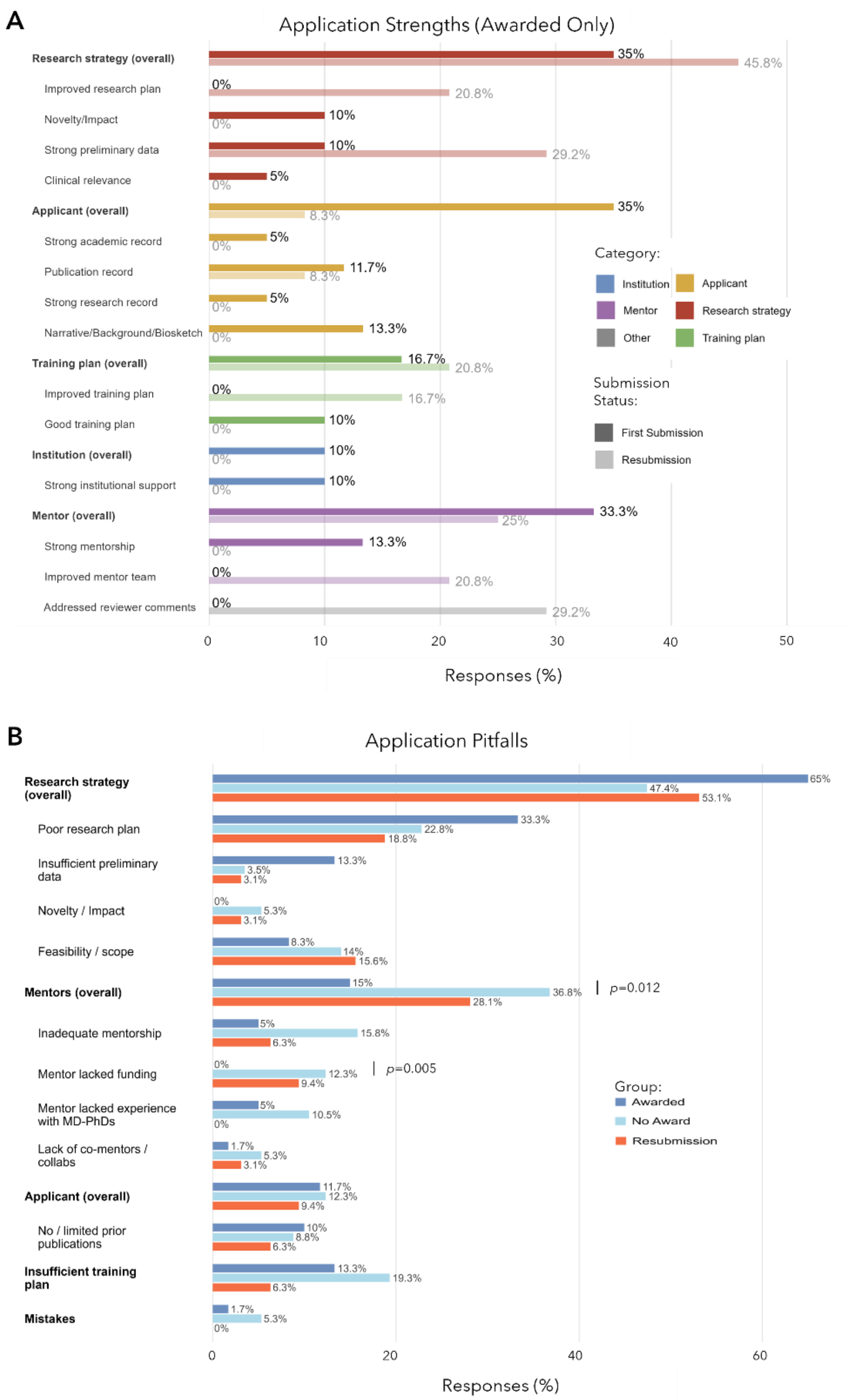

Figure S3: Application components associated with award versus no award
Univariable logistic regression analyses comparing NIH F30/F31 (predoctoral) grants that were awarded and not awarded on first submission. An odds ratio (OR)<1 indicates reduced likelihood of receiving an awarded grant, whereas OR>1 indicates increased likelihood. Statistical analysis was performed using logistic regression with Wald test. Points represent OR, lines represent 95% confidence intervals (CI), and red indicates statistical significance (p<0.05)

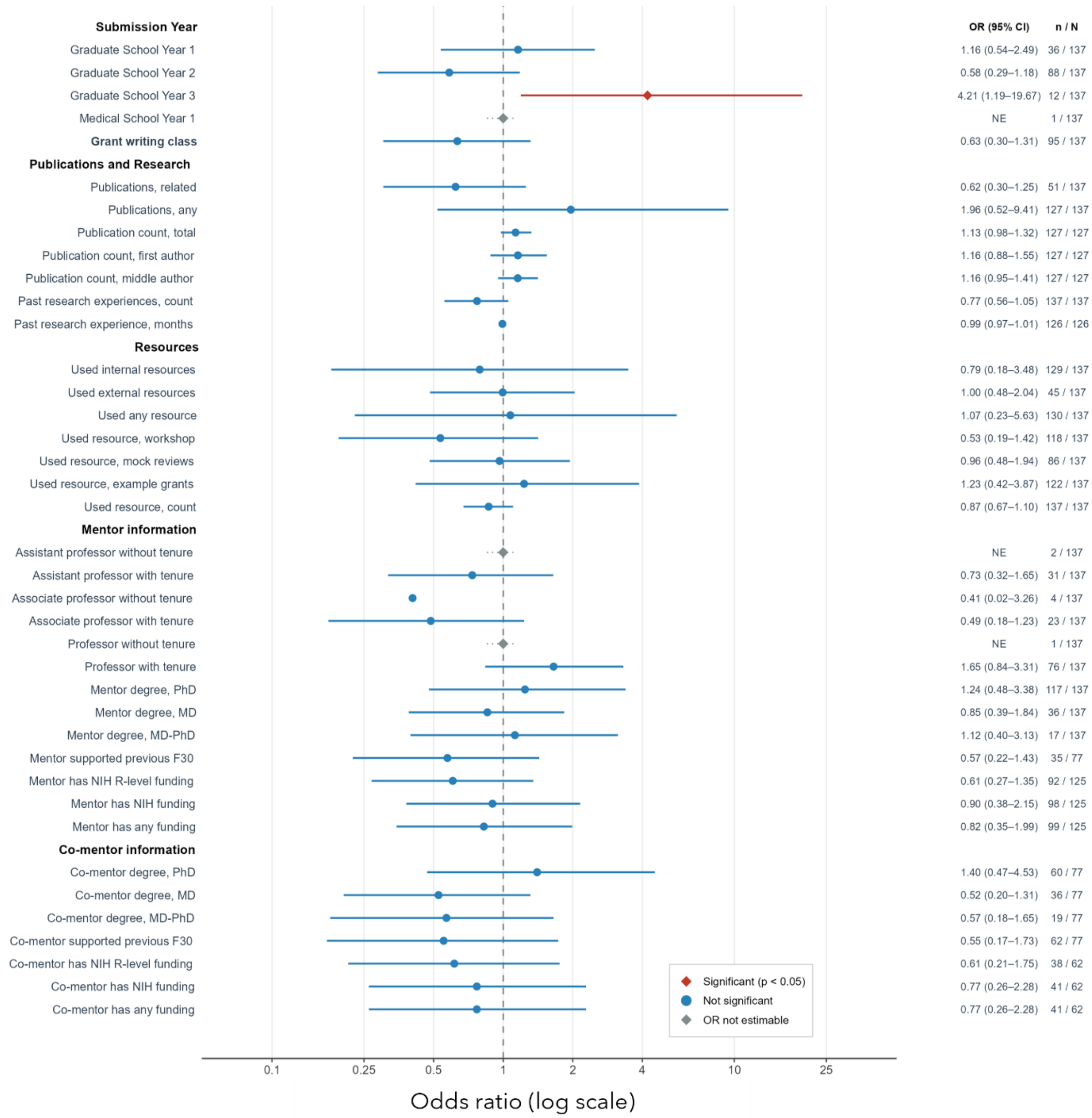

Figure S4: Major NIH F30 categorical changes made in 2025

Major categorical changes were made to pre-doctoral grant submissions in January 2025. The changes were made in an effort to minimize disparities from the suggested overinfluence of institutional status, mentor status, and applicant profile. with increased focus on the training plan, mentoring team, and trainability of the applicant. Image prepared using Perplexity.

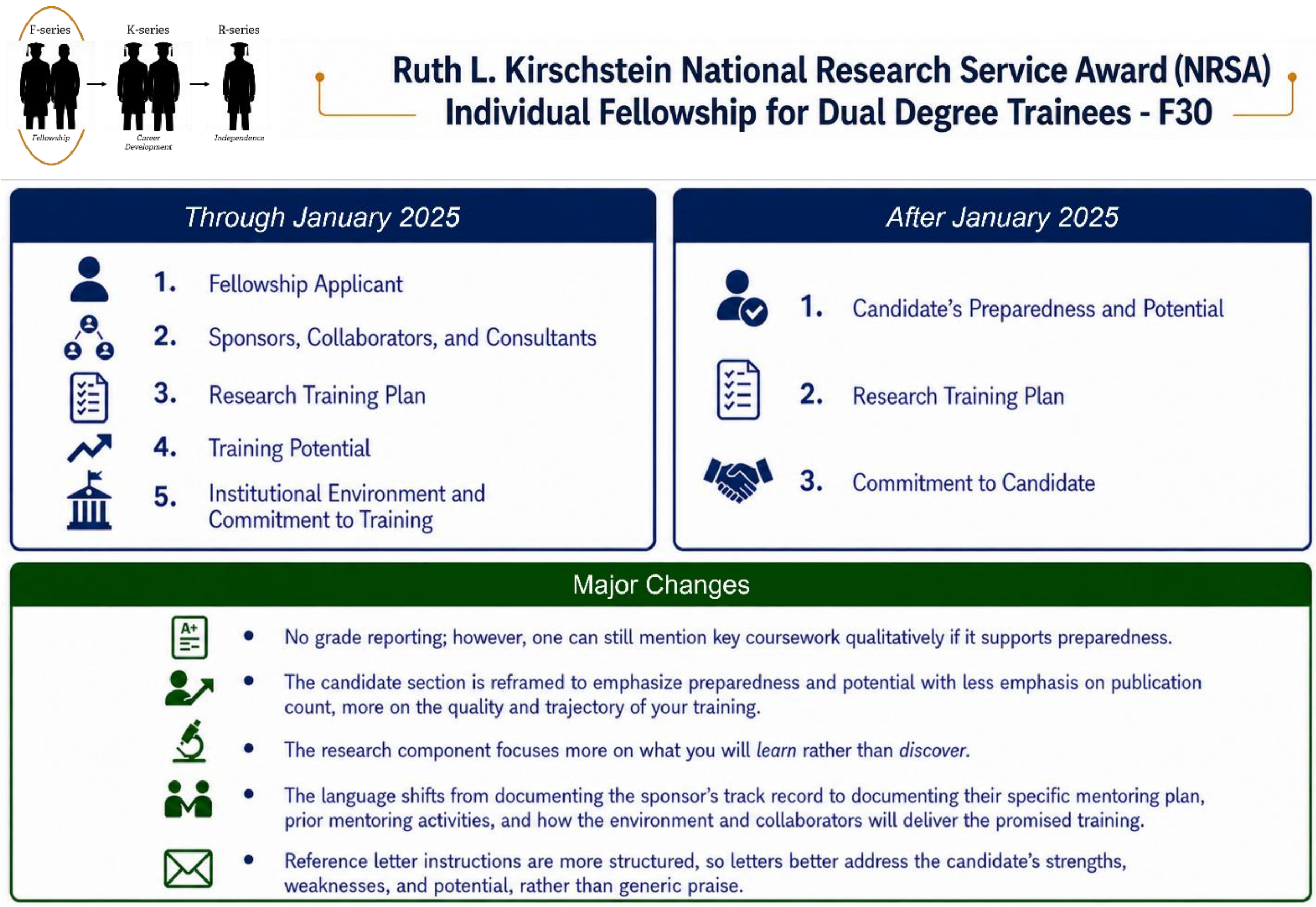

Table S1: Survey respondent demographics.

		Total n=256	Yes Submission n=137	No Submission n=119	p-val	No Award n=76	Yes Award n=61	p-val
**gender**	*man*	102	58	44	0.398	31	27	0.614
	woman	141	75	66		44	31	
	other	9	3	6		1	2	
	unspecified	4	1	3			1	
**race/ethnicity**	*white*	159	89	70		52	37	
	asian	70	35	35		17	18	
	hispanic	27	14	13		8	6	
	black	17	8	9		5	3	
	middle eastern/ north african	11	5	6		2	3	
	native american/ american indian	3	0	3		0	0	
	native hawaiian pacific islander	2	2	0		2	0	
	other	2	0	2		0	0	
	unspecified	7	4	3		2	2	
**parent degree**	*some HS*	3	0	3	0.239	0	0	0.191
	HS diploma	16	7	9		2	5	
	some college	14	9	5		4	5	
	college degree	65	37	28		23	14	
	master's degree	67	33	34		17	16	
	graduate degree (PhD)	31	15	16		10	5	
	medical degree (MD)	31	16	15		8	8	
	dual-degree (MD/DO-PhD)	6	3	3		0	3	
	other professional	14	11	3		8	3	
	other	3	3	0		3	0	
	unspecified	6	3	3		1	2	
**household income**	*<25000*	12	6	6	0.846	4	2	0.917
	25000-49999	23	10	13		6	4	
	50000-74999	27	13	14		7	6	
	75000-99999	42	21	21		11	10	
	100000-149999	58	35	23		23	12	
	150000-250000	35	19	16		10	9	
	>250000	32	18	14		9	9	
	unspecified	27	15	12		6	9	
**age at submission**	*mean*	26.86	26.6	27.7	0.017	26.7	26.53	0.646
	std dev	1.99	1.9	2.1		1.8	1.98	
	NA or unspecified	121	30	91		20	10	

Demographics were grouped by submission and award status. Submission constitutes respondents who did (Yes) or did not (No) submit an F30/F31 grant. Award constitutes respondents, who at the time of survey submitted a grant, had received (Yes) or not received (No) an award. HS = high school, NA = not applicable. The differences between census groups were compared with Chi-squared tests and Fisher exact tests when the number of responses within any subgroups was below 5. Mann-Whitney tests were used for continuous variables. Additional information such as fractions and statistical tests used is provided in the supporting data document.

Graphical abstract: Predoctoral grant writing barriers and interventions for physician-scientist trainees.

Practical interventions to help alleviate survey-identified grant writing barriers and obstacles and to improve the predoctoral grant writing experience. Image prepared using Perplexity.

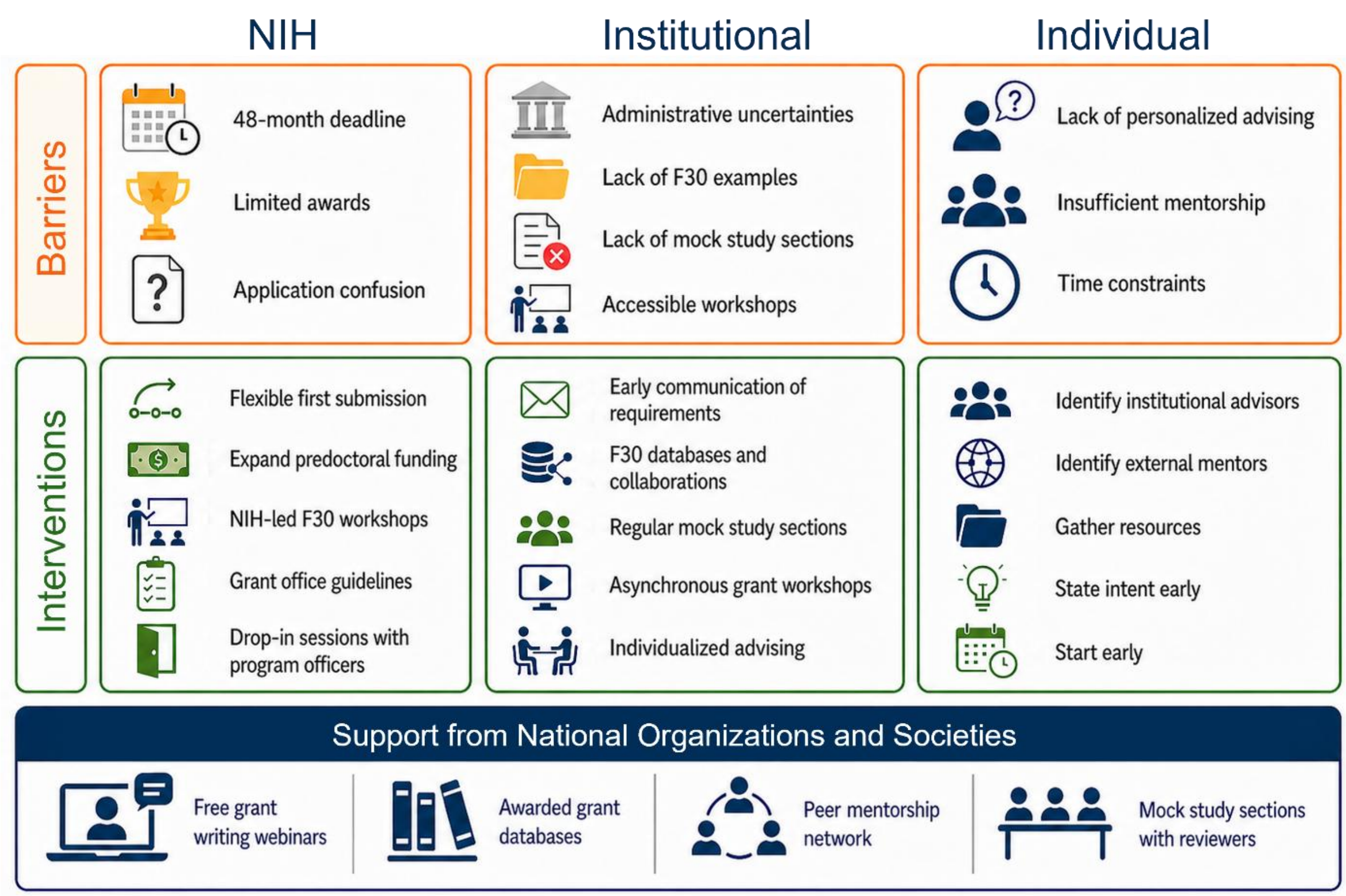

Fellowship and Grant Writing Accessibility Survey

If you are or were a dual degree candidate (MD/PhD or DO/PhD), please complete the survey below.

This survey will take approximately 10-15 minutes to complete. You do not need to have applied for a grant to participate.

For your time, you will have the option to be entered into a drawing to win a $50 gift card after submitting the survey.

Thank you.

Consent and eligibility

Assessment of Physician-Scientist Barriers to Pre-Doctoral Grant Writing

Principal Investigator/Researcher: David Arnold, MD; Brian Thomas; Cynthia Tang
IRB Reference Number: 2097283

You are being invited to take part in a research project. You must be 18 years of age or older. Your participation is voluntary, and you may stop being in this study at any time. This initiative is part of the American Physician Scientists Association's (APSA) overarching goals to increase and promote diversity, equity, and inclusion in the physician-scientist workforce. The purpose of this research project is to understand the characteristics and perceptions of pre-doctoral physician-scientist trainees toward grant writing. You are being asked to complete an anonymous survey that asks about your experience with pre-doctoral grant writing as a physician-scientist trainee. Your participation should last up to the one-time completion of this survey. This survey will take approximately 10-15 minutes to complete. For your time and effort, you will have the opportunity to optionally provide your name and email address to be included in a drawing for one of three $50 gift cards at the conclusion of the research study. Your name and email address will be collected separately from the survey and will not be linked to your survey responses. The information you provide will be kept confidential and no identifiable information will be collected. Only the research team will have access.

If you have questions about this study, you can contact the University of Missouri researcher at (314-920-3213, brian.thomas@physicianscientists.org). If you have questions about your rights as a research participant, please contact the University of Missouri Institutional Review Board (IRB) at 573-882-3181 or muresearchirb@missouri.edu. The IRB is a group of people who review research studies to make sure the rights and welfare of participants are protected. If you want to talk privately about any concerns or issues related to your participation, you may contact the Research Participant Advocacy at 888-280-5002 (a free call) or email muresearchrpa@missouri.edu.

You can ask the researcher to provide you with a copy of this consent for your records, or you can save a copy of this consent if it has already been provided to you. We appreciate your consideration to participate in this study.

By selecting "Yes" below, I consent to participate in this research study.

◯ Yes
◯ No

I am currently training in a United States MD/DO-PhD program or have completed MD/DO-PhD training in a United States MD/DO-PhD program.

◯ Yes
◯ No

Grant submission and outcomes

5% Progress

Have you ever submitted an NIH NRSA F30 or F31 individual fellowship grant?	◯ Yes<br>◯ No
What year of training did you first submit your F30/F31 (first submission)?	◯ Medical School-Year 1<br>◯ Medical School-Year 2<br>◯ Medical School-Year 3<br>◯ Medical School-Year 4<br>◯ Graduate School-Year 1<br>◯ Graduate School-Year 2<br>◯ Graduate School-Year 3<br>◯ Graduate School-Year 4<br>◯ Graduate School-Year 5<br>◯ Graduate School-Year 6+<br>◯ Other
Please specify "Other"	__________________________________

What was your target agency for your first submission?

	F30	F31
National Cancer Institute (NCI)	☐	☐
National Eye Institute (NEI)	☐	☐
National Heart, Lung, and Blood Institute (NHLBI)	☐	☐
National Human Genome Research Institute (NHGRI)	☐	☐
National Institute on Aging (NIA)	☐	☐
National Institute on Alcohol Abuse and Alcoholism (NIAAA)	☐	☐
National Institute of Allergy and Infectious Diseases (NIAID)	☐	☐
National Institute of Arthritis and Musculoskeletal and Skin Diseases (NIAMS)	☐	☐
National Institute of Biomedical Imaging and Bioengineering (NIBIB)	☐	☐
Eunice Kennedy Shriver National Institute of Child Health and Human Development (NICHD)	☐	☐
National Institute on Deafness and Other Communication Disorders (NIDCD)	☐	☐
National Institute of Dental and Craniofacial Research (NIDCR)	☐	☐
National Institute of Diabetes and Digestive and Kidney Diseases (NIDDK)	☐	☐
National Institute on Drug Abuse (NIDA)	☐	☐
National Institute of Environmental Health Sciences (NIEHS)	☐	☐
National Institute of General Medical Sciences (NIGMS)	☐	☐
National Institute of Mental Health (NIMH)	☐	☐
National Institute on Minority Health and Health Disparities (NIMHD)	☐	☐

National Institute of Neurological Disorders and Stroke (NINDS)	☐	☐
National Institute of Nursing Research (NINR)	☐	☐
National Library of Medicine (NLM)	☐	☐
Other	☐	☐
Not sure	☐	☐

Please specify "Other"

____

Was your grant awarded on the first submission?

- ◯ Yes
- ◯ No
- ◯ Currently under review or pending submission

Was your grant discussed on the first submission (i.e., within the 50th percentile or given an impact score)?

- ◯ Yes
- ◯ No

What do you believe was the biggest weakness of your application? Please be as specific as possible.

____

What do you believe was the biggest strength of your application? Please be as specific as possible.

____

Did you reapply or are you planning to reapply?

- ◯ Yes - Planning to
- ◯ Yes - Resubmission (A1)
- ◯ Yes - New submission (A0; new project)
- ◯ No

Grant resubmission and outcomes

20% Progress

Was/Is the funding mechanism (e.g., F30) and agency (e.g., NCI) the same as your first submission?

- ◯ Yes
- ◯ No

What was your new target mechanism/agency?

	F30	F31
National Cancer Institute (NCI)	☐	☐
National Eye Institute (NEI)	☐	☐
National Heart, Lung, and Blood Institute (NHLBI)	☐	☐
National Human Genome Research Institute (NHGRI)	☐	☐
National Institute on Aging (NIA)	☐	☐
National Institute on Alcohol Abuse and Alcoholism (NIAAA)	☐	☐
National Institute of Allergy and Infectious Diseases (NIAID)	☐	☐
National Institute of Arthritis and Musculoskeletal and Skin Diseases (NIAMS)	☐	☐
National Institute of Biomedical Imaging and Bioengineering (NIBIB)	☐	☐
Eunice Kennedy Shriver National Institute of Child Health and Human Development (NICHD)	☐	☐
National Institute on Deafness and Other Communication Disorders (NIDCD)	☐	☐
National Institute of Dental and Craniofacial Research (NIDCR)	☐	☐
National Institute of Diabetes and Digestive and Kidney Diseases (NIDDK)	☐	☐
National Institute on Drug Abuse (NIDA)	☐	☐
National Institute of Environmental Health Sciences (NIEHS)	☐	☐
National Institute of General Medical Sciences (NIGMS)	☐	☐
National Institute of Mental Health (NIMH)	☐	☐
National Institute on Minority Health and Health Disparities (NIMHD)	☐	☐

National Institute of Neurological Disorders and Stroke (NINDS)	☐	☐
National Institute of Nursing Research (NINR)	☐	☐
National Library of Medicine (NLM)	☐	☐
Not sure	☐	☐
Other	☐	☐

Please specify "Other"

____

What year of training did you re-submit your F30/F31?

◯ Medical School-Year 1
◯ Medical School-Year 2
◯ Medical School-Year 3
◯ Medical School-Year 4
◯ Graduate School-Year 1
◯ Graduate School-Year 2
◯ Graduate School-Year 3
◯ Graduate School-Year 4
◯ Graduate School-Year 5
◯ Graduate School-Year 6+
◯ Other

Please specify "Other"

____

Was your grant awarded upon resubmission?

◯ Yes
◯ No
◯ Currently under review or pending submission

Was your grant discussed upon resubmission (i.e., within 50th percentile or given an impact score)?

◯ Yes
◯ No

What do you believe was the biggest driving factor in turning your resubmission into a successful application?

____

What do you believe was your biggest pitfall to your resubmission? Please be as specific as possible.

____

Have you applied for another non-NIH fellowship during your dual degree training? These can include internal (within University) or external fellowships.

◯ Yes
◯ No

What is the funding agency, institution, and mechanism? Please indicate whether these were internal or external fellowships.

____

Do you plan to apply for an F30, F31, or equivalent grant?

◯ Yes
◯ No

What is your planned target mechanism/agency?

	F30	F31
National Cancer Institute (NCI)	☐	☐
National Eye Institute (NEI)	☐	☐
National Heart, Lung, and Blood Institute (NHLBI)	☐	☐
National Human Genome Research Institute (NHGRI)	☐	☐
National Institute on Aging (NIA)	☐	☐
National Institute on Alcohol Abuse and Alcoholism (NIAAA)	☐	☐
National Institute of Allergy and Infectious Diseases (NIAID)	☐	☐
National Institute of Arthritis and Musculoskeletal and Skin Diseases (NIAMS)	☐	☐
National Institute of Biomedical Imaging and Bioengineering (NIBIB)	☐	☐
Eunice Kennedy Shriver National Institute of Child Health and Human Development (NICHD)	☐	☐
National Institute on Deafness and Other Communication Disorders (NIDCD)	☐	☐
National Institute of Dental and Craniofacial Research (NIDCR)	☐	☐
National Institute of Diabetes and Digestive and Kidney Diseases (NIDDK)	☐	☐
National Institute on Drug Abuse (NIDA)	☐	☐
National Institute of Environmental Health Sciences (NIEHS)	☐	☐
National Institute of General Medical Sciences (NIGMS)	☐	☐
National Institute of Mental Health (NIMH)	☐	☐
National Institute on Minority Health and Health Disparities (NIMHD)	☐	☐

National Institute of Neurological Disorders and Stroke (NINDS)	☐	☐
National Institute of Nursing Research (NINR)	☐	☐
National Library of Medicine (NLM)	☐	☐
Equivalent non-NIH	☐	☐
Not sure	☐	☐
Other	☐	☐

Please specify "Other"

____

Please specify "non-NIH equivalent"

____

Why will you not apply or re-submit? Please select all that apply.

- ☐ Do not have the resources to submit a successful application
- ☐ Do not have enough time to complete a proposal
- ☐ Do no have a strong research agenda (i.e., no defined research project)
- ☐ Am not interested in an academic research path after graduation
- ☐ No previous knowledge of grants and fellowships or not sure of the application process
- ☐ Too competitive
- ☐ Research does not align with an NIH institutional (e.g., NCI, NIAID, etc.)
- ☐ Already have other fellowship funding and/or scholarships
- ☐ Personal or family related issues
- ☐ Unsupportive mentor
- ☐ Unsupportive institution
- ☐ Not eligible, I waited too long from original submission to resubmit
- ☐ Not eligible, It is too late to submit or resubmit (F30 only; regarding 48 month after matriculation limit)
- ☐ Not eligible, Other
- ☐ Other or prefer to self describe
- ☐ Prefer not to say

Please specify "Other"

____

Please specify what resources were unavailable. Select all that apply.

- ☐ Grant writing course, workshop, or information session
- ☐ Mock review session
- ☐ Examples of successful F30/F31 grants
- ☐ Faculty mentor
- ☐ Peer mentor (e.g. student who previously submitted an F grant)
- ☐ Other

Please specify "Other"

____

If you had access to additional resources would you consider submitting an application?

◯ Yes
◯ No

If you had access to additional resources would you consider resubmitting an application?

◯ Yes
◯ No

Any additional comments?

____

Resources, Utilization, and Perceptions

40% Progress

Please select all that apply.

	Grant writing workshop specific to F30 or F31	Grant writing information session specific to F30 or F31	Additional advising for F30, F31	Mock review session	Examples of successful F30, F31 applications	None	Not sure	Not applicable
Which of these resources does your MD/DO-PhD program offer:	☐	☐	☐	☐	☐	☐	☐	☐
Which of these resources does your institution (e.g., programs outside of your dual degree program such as graduate only) offer:	☐	☐	☐	☐	☐	☐	☐	☐
Which of these program or institutional (internal) resources did you utilize or are you currently utilizing:	☐	☐	☐	☐	☐	☐	☐	☐
Which of these resources have you used from outside (external) your program or institution:	☐	☐	☐	☐	☐	☐	☐	☐
What resources did/do you find most useful:	☐	☐	☐	☐	☐	☐	☐	☐
What resources do you think would be useful for F30/F31 grant writing that you did/do not have access to:	☐	☐	☐	☐	☐	☐	☐	☐

What was the name of the external grant writing workshop?

____

Did/do you have access to peers who have previously submitted an F30/F31? Please select all that apply.

☐ Yes, successful (funded) peer applicants
☐ Yes, unsuccessful (not funded) peer applicants
☐ Yes, unsure if peer applicants were successful or not OR they were waiting on scores
☐ No

Did/will you use any ADDITIONAL resources for F30/F31 grant writing? If so, please describe.

____

Are there any ADDITIONAL resources that you would find useful for F30/F31 grant writing that you did/do not have access too? If so, please describe.

____

Please rank the following as a perceived OBSTACLE to applying for an F award?

	1 - Not an obstacle	2 - Minor obstacle	3 - Moderate obstacle	4 - Significant obstacle
Effort required to complete	○	○	○	○
Finding collaborators	○	○	○	○
Sponsor or co-sponsor support	○	○	○	○
Access to resources (e.g., access to example F30's, grant review sessions, etc.))	○	○	○	○

Were/was there any ADDITIONAL perceived obstacles to applying for an F award? If so, please describe.

____

Please rank the following as a perceived BENEFIT to applying for an F award?

	1 - Not a benefit	2 - Minor benefit	3 - Moderate benefit	4 - Significant benefit
Grant writing experience	◯	◯	◯	◯
Financial support	◯	◯	◯	◯
Establishing a funding track record for future grant applications	◯	◯	◯	◯
Strengthening CV for future career or residency applications	◯	◯	◯	◯

Were/was there any ADDITIONAL perceived benefits to applying for an F award? If so, please describe.

____

Applicant information.

For those who are no longer dual degree trainees (e.g., resident, faculty, etc.), please answer to the best of your ability for when you were in enrolled in your program unless otherwise noted.

Current Classification	◯ MD-PhD Student<br>◯ DO-PhD Student<br>◯ Resident/Fellow with prior MD/DO-PhD training<br>◯ Post-Doc with prior MD/DO-PhD training<br>◯ Faculty with prior MD/DO-PhD training<br>◯ Other
Please specify "Other"	__________________________
Current Training Stage (check all that apply)	☐ Medical School-Year 1<br>☐ Medical School-Year 2<br>☐ Medical School-Year 3<br>☐ Medical School-Year 4<br>☐ Graduate School-Year 1<br>☐ Graduate School-Year 2<br>☐ Graduate School-Year 3<br>☐ Graduate School-Year 4<br>☐ Graduate School-Year 5<br>☐ Graduate School-Year 6+<br>☐ Internship<br>☐ Residency<br>☐ Fellowship<br>☐ Post-Doctoral Research<br>☐ Faculty<br>☐ Other
Please specify "Other"	__________________________
Since your F30/F31 submission, have you applied for a career development (K) award (e.g., K08, K99, etc.) or R01? Please select all that apply.	☐ Yes, K - awarded<br>☐ Yes, K - not awarded<br>☐ Yes, R01 - awarded<br>☐ Yes, R01 - not awarded<br>☐ No<br>☐ Prefer not to say
Approximately how many years was it from your last F30/F31 submission to time of K award?	__________________________
Approximately how many years was it from your last F30/F31 submission to time of R01 award?	__________________________
MD/DO-PhD Program Type	◯ NIH funded MSTP<br>◯ Non-NIH funded MD/DO-PhD program<br>◯ Other (describe below)
Does your program provide tuition support?	◯ Yes<br>◯ No
Does your program provide a stipend?	◯ Yes<br>◯ No

Research field of study	◯ Basic<br>◯ Translational<br>◯ Clinical<br>◯ Social<br>◯ Behavioral<br>◯ Political<br>◯ Other
Please specify "Other"	__________________________________
What career sector do you foresee yourself in after MD/DO-PhD training? Please check the MOST likely sector. (Limit 2). If you have already completed the dual degree program, please select your career sector.	☐ Academia - Research<br>☐ Academia - Non-Research<br>☐ Industry<br>☐ Government<br>☐ Private Practice<br>☐ Hospitalist<br>☐ Consulting<br>☐ Other
Please specify "Other"	__________________________________

Applicant experience

60% Progress

How many full-time (>30 h/week) research experiences have you had before starting your PhD training?	◯ 0<br>◯ 1<br>◯ 2<br>◯ 3<br>◯ 4+
How many months of full-time (>30 hrs/week) research have you done before starting your PhD training?	__________________________________
Were you supported on an institutional T32 NIH grant at any point PRIOR to applying for the F30/31?	◯ Yes<br>◯ No
Did you take a grant writing course at any point during your dual degree training?	◯ Yes - before first submission<br>◯ Yes - before re-submission (after first submission)<br>◯ Yes - after all submissions<br>◯ No
Did you have any publications at the time of your FIRST F30/F31 submission? Please select all that apply.	☐ Yes, related to proposed project - first author<br>☐ Yes, related to proposed project middle author<br>☐ Yes, unrelated to proposed project<br>☐ No<br>☐ No, but plan to upon submission or resubmission<br>☐ Not sure

How many publications did you have for each at your FIRST submission? (Related: similar subject area to proposed project)

	0	1	2	3	4	5+
Related - first author	○	○	○	○	○	○
Related - middle author	○	○	○	○	○	○
Unrelated - first author	○	○	○	○	○	○
Unrelated - middle author	○	○	○	○	○	○

Did you have any ADDITIONAL publications at the time of your F30/F31 RESUBMISSION? Please select all that apply.

Do not include publications from first submission.

- ☐ Yes, related to proposed project - first author
- ☐ Yes, related to proposed project middle author
- ☐ Yes, unrelated to proposed project
- ☐ No
- ☐ No, but plan to upon submission or resubmission
- ☐ Not sure

How many ADDITIONAL publications did you have for each at RESUBMISSION? (Related: similar subject area to proposed project).

Do not include publications from first submission.

	0	1	2	3	4	5+
Related - first author	◯	◯	◯	◯	◯	◯
Related - middle author	◯	◯	◯	◯	◯	◯
Unrelated - first author	◯	◯	◯	◯	◯	◯
Unrelated - middle author	◯	◯	◯	◯	◯	◯

Did you have any track record of self-applied successful funding aside from MD-PhD matriculation fellowship at the time of your FIRST F30/F31 submission? Please select all that apply.

- ☐ Yes, another fellowship - undergraduate
- ☐ Yes, another fellowship - graduate
- ☐ Yes, travel award
- ☐ Yes, other
- ☐ No

Please specify "Other"

____

Did you have any ADDITIONAL track record of self-applied successful funding aside from MD-PhD matriculation fellowship at the time of your FINAL F30/F31 submission? Please select all that apply.

Do not include other funding from first submission.

- ☐ Yes, another fellowship - undergraduate
- ☐ Yes, another fellowship - graduate
- ☐ Yes, travel award
- ☐ Yes, other
- ☐ No

Please specify "Other"

____

Mentor/sponsor information

80% Progress

How much was your main mentor (sponsor) involved in grant preparation?

◯ 0 - Not at all
◯ 1 - Minimally
◯ 2 - Moderately
◯ 3 - Extensively

What is the title of your main mentor (sponsor)?

◯ Assistant Professor (Non-Tenure Track)
◯ Associate Professor (Non-Tenure Track)
◯ Professor (Non-Tenure Track)
◯ Assistant Professor (Tenure Track)
◯ Associate Professor (Tenure Track)
◯ Professor (Tenure Track)
◯ Other

Please specify "Other"

____

What degree's did you main mentor hold? Please select all that apply.

☐ PhD ☐ MD ☐ DO
☐ MPH ☐ MS ☐ Other

Please specify "Other"

____

Did your main mentor (sponsor) have NIH funding during your MD/DO-PhD training

◯ Yes ◯ No ◯ Not sure

Was your main mentors (sponsor) fundings in the same agency as your F application/award? What was the mechanism of funding?

☐ Yes - R01 ☐ Yes - R01-like (non-NIH; e.g., DoD, NSF, NASA) ☐ Yes - R21
☐ Yes - R56 ☐ Yes - Other NIH
☐ Yes - Other non-NIH ☐ No - R01
☐ No - R01-like (non-NIH; e.g., DoD, NSF, NASA) ☐ No - R21 ☐ No - R56
☐ No - Other NIH ☐ No - Other non-NIH
☐ Not sure ☐ Do not wish to share

Please specify "Other"

____

Has your main mentor (sponsor) previously supported an successful or unsuccessful F30/31 award application?

◯ Yes - successful ◯ Yes - unsuccessful
◯ Yes - not sure ◯ No
◯ Not sure ◯ Do not wish to share

Did you have a secondary mentor (co-sponsor)?

◯ Yes
◯ No

How much was your secondary mentor (co-sponsor) involved in grant preparation?

◯ 0 - Not at all
◯ 1 - Minimally
◯ 2 - Moderately
◯ 3 - Extensively

What is the title of your secondary mentor (co-sponsor)?	◯ Assistant Professor (Non-Tenure Track)<br>◯ Associate Professor (Non-Tenure Track)<br>◯ Professor (Non-Tenure Track)<br>◯ Assistant Professor (Tenure Track)<br>◯ Associate Professor (Tenure Track)<br>◯ Professor (Tenure Track)<br>◯ Other
Please specify "Other"	__________________________________
What degree's did you secondary mentor (co-sponsor) hold? Please select all that apply.	☐ PhD ☐ MD ☐ DO<br>☐ MPH ☐ MS ☐ Other
Please specify "Other"	__________________________________
Did your secondary mentor (co-sponsor) have NIH funding during your MD/DO-PhD training	◯ Yes ◯ No ◯ Not sure
Was your secondary mentors (co-sponsor) fundings in the same agency as your F application/award? What was the mechanism of funding?	☐ Yes - R01 ☐ Yes - R01-like (non-NIH; e.g., DoD, NSF, NASA) ☐ Yes - R21<br>☐ Yes - R56 ☐ Yes - Other NIH<br>☐ Yes - Other non-NIH ☐ No - R01<br>☐ No - R01-like (non-NIH; e.g., DoD, NSF, NASA) ☐ No - R21 ☐ No - R56<br>☐ No - Other NIH ☐ No - Other non-NIH<br>☐ Not sure ☐ Do not wish to share
Please specify "Other"	__________________________________
Has your secondary mentor (co-sponsor) previously supported a successful or unsuccessful F30/31 award application?	◯ Yes - successful ◯ Yes - unsuccessful<br>◯ Yes - not sure ◯ No<br>◯ Not sure ◯ Do not wish to share

Demographics

90% Progress

Age at time of final F30/F31 submission	__________________________________
Gender	☐ Man<br>☐ Woman<br>☐ Non-binary/non-conforming/third gender<br>☐ Transgender<br>☐ Genderqueer<br>☐ Prefer not to say<br>☐ Prefer to self describe
Please specify	__________________________________
Race/Ethnicity	☐ American Indian or Native American<br>☐ Asian<br>☐ Black or African American<br>☐ Hispanic or Latinx/a/o/e<br>☐ Middle Eastern or North African<br>☐ Native Hawaiian or Pacific Islander<br>☐ White<br>☐ Prefer not to say<br>☐ Prefer to self-describe
Please specify	__________________________________
Highest degree complete by your parents	◯ Some HS<br>◯ HS diploma or equivalent (GED)<br>◯ Some college<br>◯ College degree<br>◯ Graduate degree (Masters)<br>◯ Graduate degree (PhD)<br>◯ Medical degree (MD or DO)<br>◯ Dual degree (MD-PhD or DO-PhD)<br>◯ Other professional (DVM, PT, OT, Doctor of Nursing, etc.)<br>◯ Other<br>◯ Prefer not to say
Please specify "Other"	__________________________________
Yearly household income during childhood	◯ < $25,000<br>◯ $25,000 to 49,999<br>◯ $50,000 to 74,999<br>◯ $75,000 to 99,999<br>◯ $100,000 to 149,999<br>◯ $150,000 to 250,000<br>◯ > $250,000<br>◯ Prefer not to say
Please include any additional information that you would like to share about your grant writing experience.	____
